# Spontaneous healing of *Mycobacterium ulcerans* disease in Australian patients

**DOI:** 10.1371/journal.pntd.0007178

**Published:** 2019-02-19

**Authors:** Daniel P. O’Brien, Adrian Murrie, Peter Meggyesy, Jonathan Priestley, Avinash Rajcoomar, Eugene Athan

**Affiliations:** 1 Department of Infectious Diseases, Barwon Health, Geelong, Victoria, Australia; 2 Department of Medicine and Infectious Diseases, Royal Melbourne Hospital, University of Melbourne, Melbourne, Victoria, Australia; 3 Sorrento Medical Centre, Sorrento, Victoria, Australia; 4 South Coast Medical, Blairgowrie, Victoria, Australia; 5 Ocean Grove Medical Center, Ocean Grove, Victoria, Australia; Swiss Tropical and Public Health Institute, SWITZERLAND

## Abstract

**Background:**

*Mycobacterium ulcerans* causes necrotising infections of skin and soft tissue mediated by the polyketide exotoxin mycolactone that causes cell apoptosis and immune suppression. It has been postulated that infection can be eradicated before the development of clinical lesions but spontaneous resolution of clinical lesions has been rarely described.

**Methodology/Principal findings:**

We report a case series of five Australian patients who achieved healing of small *M*. *ulcerans* lesions without antibiotics or surgery. The median age of patients was 47 years (IQR 30–68 years) and all patients had small ulcerative lesions (median size 144mm^2^, IQR 121-324mm^2^). The median duration of symptoms prior to diagnosis was 90 days (IQR 90–100 days) and the median time to heal from diagnosis without treatment was 68 days (IQR 63–105 days). No patients recurred after a median follow-up of 16.6 months (IQR 16.6–17.9 months) from the development of symptoms and no patients suffered long-term disability from the disease.

**Conclusions:**

We have shown that healing without specific treatment can occur for small ulcerated *M*. *ulcerans* lesions suggesting that in selected cases a robust immune response alone can cure lesions. Further research is required to determine what lesion and host factors are associated with spontaneous healing, and whether observation alone is an effective and safe form of management for selected small *M*. *ulcerans* lesions.

## Author summary

*Mycobacterium ulcerans* causes a destructive infection of skin and soft tissue known as Buruli ulcer that when severe can lead to serious long-term deformity and disability. It is currently not well documented whether people with *Mycobacterium ulcerans* disease can cure themselves without treatment. In our study we describe five people with small ulcers who cured their disease without specific medical or surgical treatment. This suggests that a proportion of people can develop an immune response sufficient enough to eradicate the disease without the help of medical intervention. This is an important step, as recognition of this possibility provides important further insights into the human immune response against the disease. It also opens the possibility to further studies that may determine characteristics of the organism and hosts that favour spontaneous healing of lesions. This knowledge may in turn improve efforts to prevent and control the disease which are currently lacking.

## Introduction

*Mycobacterium ulcerans* causes a necrotising infection of skin and soft tissue known as Buruli ulcer (BU). If untreated it usually progresses, can result in major tissue destruction and be complicated by bone or joint infection.[1] In severe cases it may require plastic and reconstructive surgery and result in long-term disability.[2] The pathogenesis of *M*. *ulcerans* is mediated by a plasmid produced polyketide exotoxin called mycolactone which causes tissue destruction by inducing cell apoptosis.[3] It also allows persisting infection to develop by inhibiting dendritic cell function and secondarily T-cell activation,[4,5] as well as reducing the function of monocytes and macrophages by inhibiting cytokine production including tissue necrosis factors and gamma interferon.[6,7]

It has been postulated that infection can be eradicated before the development of clinical lesions,[8] and a partial protective effect of BCG in humans has been reported.[9] If true, these observations suggest that the hosts immune response can be protective against the development of BU, thought to be mediated via a protective T-helper-1 (TH1) cell mediated immune response.[10] Spontaneous resolution without medical or surgical treatment of clinical lesions in humans has been rarely reported.[11–13] Furthermore, in one of these studies involving five lesions from Africa the lesions were not bacteriologically confirmed to be *M*. *ulcerans*,[12] and in another study of a single lesion from Australia the lesion was surgically excised.[11] Recently Marion et al reported a case from Benin where a small nodular *M*. *ulcerans* lesion healed without medical or surgical intervention, as well as a small group of patients with active large *M*. *ulcerans* lesions who had separate scars suggestive of previously spontaneously healed large *M*. *ulcerans* lesions.[13] It is also unknown how often spontaneous resolution occurs and the factors associated with it. Therefore treatment is recommended for all *M*. *ulcerans* lesions.[14,15] The recommended first-line treatment is combination antibiotics for 8 weeks which is highly effective.[16,17] Wide surgical excision without antibiotics can be performed, with cure rates of greater than 90% if reserved for selected lesions with no risk factors for recurrence.[14,18] In a study from Africa, local heat application without antibiotics achieved high initial wound healing rates, but 18% of patients developed a recurrent lesion within 2 years.[19]

The endemic region of Victoria, Australia, is facing a worsening epidemic of *M*. *ulcerans* disease, with control efforts hampered by the limited understanding of transmission mechanisms to humans as well as the risk and mechanisms of disease development following exposure and infection.[20] Identifying that some patients can heal their disease without treatment, and the study of the factors that allowed them to do so, may provide insights that could aid the improved control of *M*. *ulcerans* disease. In this paper we report a case series of five Australian patients who achieved healing of their confirmed *M*. *ulcerans* lesions without recommended antibiotic regimens or surgery.

## Methods

This was an observational study of routinely collected data from a clinical cohort of *M*. *ulcerans* patients managed at Barwon Health as previously described.[21] All patients were from the *M*. *ulcerans* endemic region of the Mornington and Bellarine Peninsulas in Victoria, Australia.[22] They were all diagnosed in 2017 on the basis of a positive *IS2404* PCR for *M*. *ulcerans*.[23] The size of the lesion was determined by measuring with a ruler the diameter of induration in millimetres and calculating the surface area in millimetres squared. Patients were followed up on a 2 to 4 week basis until wound healing was achieved, and then at the end of the study period. Data on the type and frequency of wound dressings was not collected, although due to the small size of lesions, wound dressings were frequently not administered.

### Results

The five cases of *M*. *ulcerans* disease all occurred in adults as single small ulcerative lesions ranging from 16 to 858 mm^2^ in size (median size 144mm^2^, IQR 121-324mm^2^). (Table 1). The median age of patients was 47 years (IQR 30–68 years) and there were 3 males and two females. No patients were known to be immune suppressed or have diabetes. HIV testing was not performed. The median duration of symptoms prior to diagnosis was 90 days (IQR 90–100 days). In one case an acid-fast bacilli (AFB) stain and culture for *M*. *ulcerans* were also positive.

**Table 1:** Characteristics of five *M*. *ulcerans* disease lesions that spontaneously healed.

Patient Number	Age at diagnosis (years)	Duration of symptoms at diagnosis (days)	Gender	WHO Category	Lesion size at baseline (mm^2^)	Lesion site	Type of lesion	Diagnostic specimen	AFB Smear	PCR	Culture	Time to heal from diagnosis (days)	Follow-up time since symptoms developed (months)
1	68	90	Male	1	324	ELBOW	ULCER	Swab	Positive	Positive	Positive	105	22.3
2	28	100	Male	1	858	BUTTOCK	ULCER	Biopsy	ND	Positive	ND	63	17.9
3	69	90	Male	1	121	LEG	ULCER	Swab	ND	Positive	ND	68	16.6
4	47	120	Female	1	144	FOOT	ULCER	Swab	Negative	Positive	Negative	120	16.6
5	30	84	Female	1	16	LEG	ULCER	Swab	ND	Positive	ND	34	13.7

ND: Not done

### Ethics statement

All patients gave informed oral consent to be managed with observation only and ethics approval for the study was provided by the Barwon Health Ethics Committee. All data were analysed anonymously.

**Fig 1:**
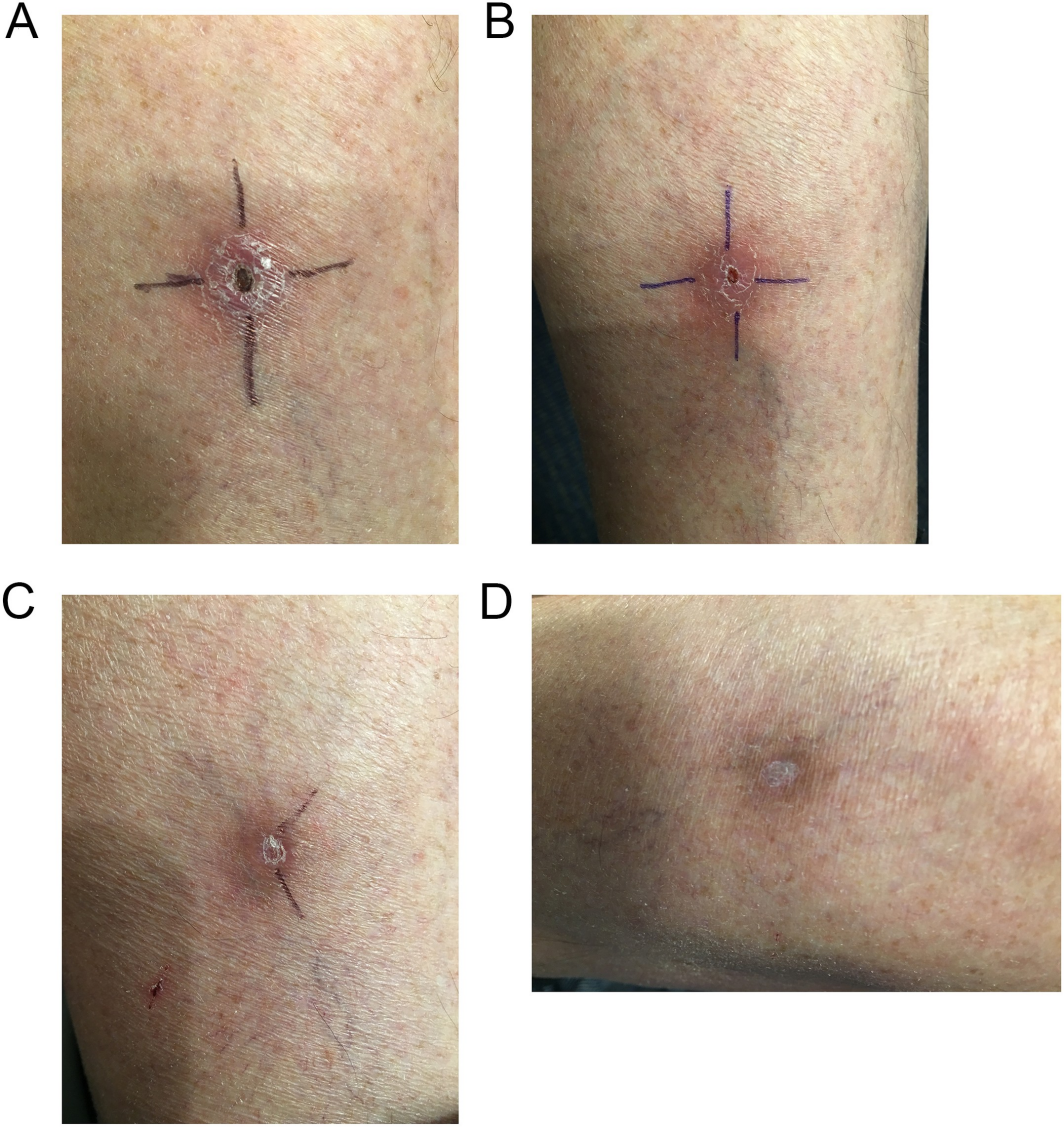
Spontaneous healing of *M*. *ulcerans* lesion left calf; a) at diagnosis, b) 2 months post diagnosis, c) 3 months post diagnosis, and d) 7 months post diagnosis.

**Fig 2:**
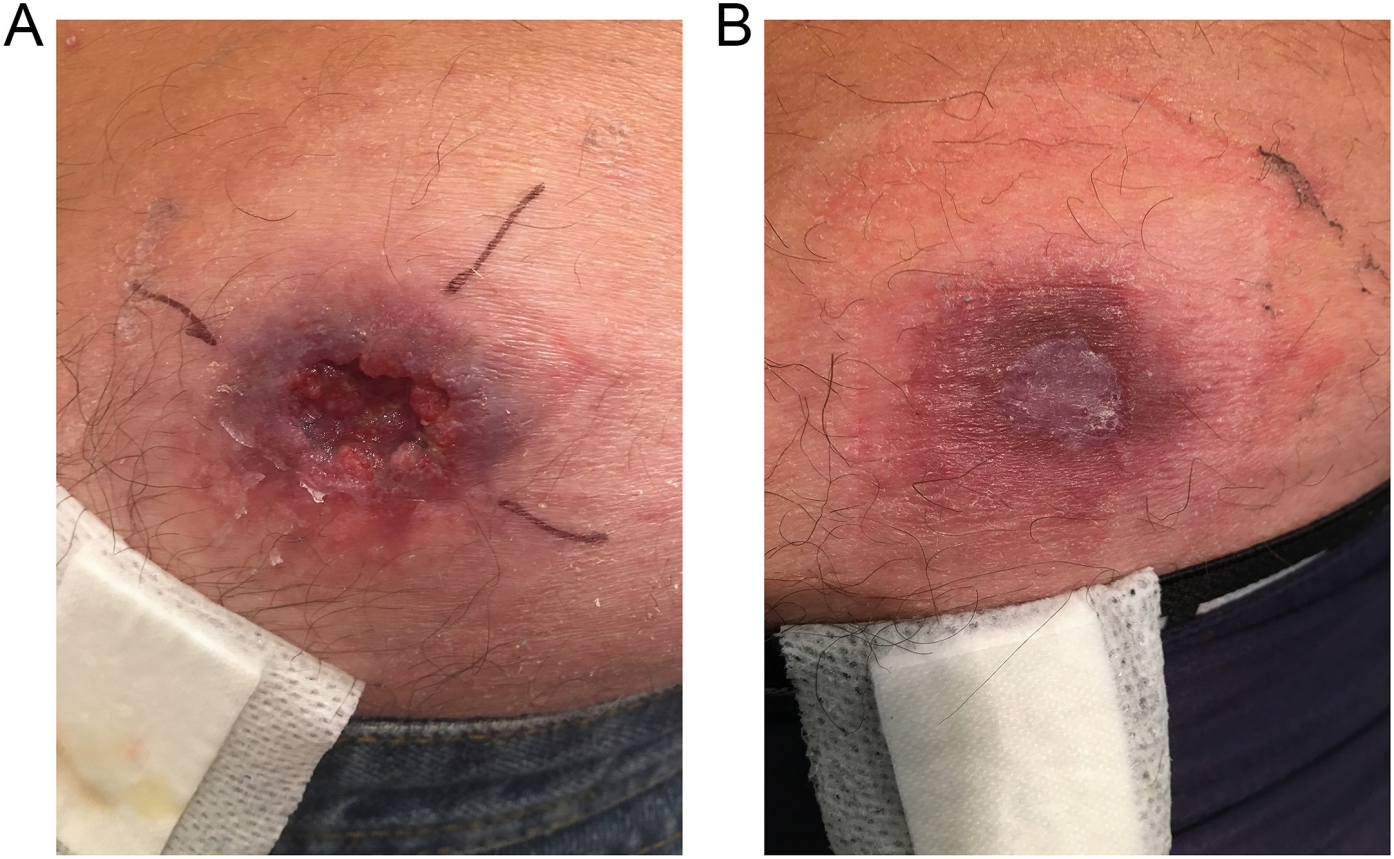
Spontaneous healing of *M*. *ulcerans* lesion buttock; a) at diagnosis, and b) 2 months post diagnosis.

Patient # 2 had an incisional biopsy but no other specific treatment. No other patients received recommended antibiotics or surgical treatment due to patient choice—in all 5 cases due to a reluctance to risk the toxicity of antibiotics or to undergo surgery in view of the small size of their *M*. *ulcerans* lesion. No patients were given heat treatment. The median time to heal from diagnosis was 68 days (IQR 63–105 days). (Figs 1 and 2) No patients had recurred after a median follow-up of 16.6 months (IQR 16.6–17.9 months) from the development of symptoms. No patients suffered long-term disability from the disease.

One patient in the cohort, a 37 year old male diagnosed in 2017 by *IS2404* PCR after 120 days of symptoms, was initially managed with observation alone but was changed to active treatment after 49 days of observation following an increase in size of his lesion from 154 mm^2^ to 340 mm^2^. He was subsequently cured with 4 weeks of rifampicin and clarithromycin antibiotic treatment combined with a surgical curette and did not suffer any long-term disability.

## Discussion

This case series demonstrates that there are a proportion of patients with confirmed small ulcerative *M*. *ulcerans* lesions that spontaneously heal without specific antibiotic or surgical treatment. In our case series it is likely that all patients have been cured of their disease as they were followed for at least 14 months from the development of symptoms without evidence of relapse. We have previously demonstrated in Australian patients that disease relapse is rare more than 12 months following diagnosis and treatment.[24]

This suggests that in selected patients, the development of host immunity following the development of clinical disease may be effective in curing lesions. This presumably results from the host’s immune protection overcoming the immune suppressive effects of the mycolactone. This may relate to the development of a more robust TH1 immune response. The importance of the TH1 immune response in combating *M*. *ulcerans* disease is suggested by the fact that the expression level of gamma-interferon is inversely correlated with the severity of M. ulcerans lesions,[25] and gamma-interferon knockout mice developed more severe *M*. *ulcerans* disease with a greater numbers of organisms.[10]

It is notable that all our patients had symptoms for at least 84 days prior to diagnosis yet the lesions had remained small (<900mm^2^). This suggests lesions that have not progressed significantly in the first three months are exhibiting a degree of immune control that may allow spontaneous healing to occur. In addition, small lesions may have a lower number of organisms with less mycolactone production to inhibit the immune system, favouring the host’s immune response against the organism’s persistence. Observed factors in our cases series that may favour spontaneous resolution include small lesion size after at least approximately 90 days of symptoms, the lack of associated co-morbidities such as diabetes or malignancy that may impair the host’s immune response, and ulcerative lesions which allow the discharge of necrotic material that may contain live organisms and mycolactone. Our study is limited by the lack of further immunological testing of the host and biological testing of isolates and therefore we suggest further research be performed to examine host and pathogen factors associated with spontaneous healing of *M*. *ulcerans* disease. This will hopefully further enhance the understanding of human immune function against the organism which may in turn allow improved treatment of the disease. Furthermore, it may provide insights that allow the development of interventions that prevent disease post exposure, such as vaccination, an area for which the current lack of knowledge hampers disease control efforts.[20]

It is important to acknowledge that we have not performed a controlled trial comparing observation alone to active treatment of small ulcerative *M*. *ulcerans* lesions and therefore we cannot make conclusions about the safety and effectiveness of this approach as a mode of management. It is also important to understand that all patients had very small lesions that were not at risk of severe complications or disability without specific treatment—for larger lesions immediate antibiotic treatment is important to achieve optimal outcomes, and although spontaneous healing may be possible in severe lesions over a lengthy period, in most patients irreversible physical impairment occurs as a consequence.[13] Nevertheless, the recognition that some small lesions can resolve spontaneously suggests that further studies could be performed to determine the true prevalence of spontaneously healing small *M*. *ulcerans* lesions and whether there is the potential for treating clinicians to safely and effectively employ close observation for similar small lesions, rather than immediate antibiotic or surgical treatment. Additionally, it would be important to determine what lesion and host factors favour this approach. Observation alone has the advantage of avoiding the potential toxicity of antibiotic treatment which results in serious adverse effects in more than 20% of treated Australian patients.[26] Although surgery alone can be an effective option for small lesions[18] this usually involves a financial cost and is not always easily accessible. Importantly, observation alone as a mode of management for small lesions would need to be evaluated in settings with lower resources and more isolated populations where close monitoring may be less feasible, increasing the risk of undetected disease progression.

In conclusion, healing without specific treatment can occur for some small ulcerated *M*. *ulcerans* lesions in Australian patients suggesting that in selected cases a robust immune response alone can cure lesions. Further research is required to determine what lesion and host factors are associated with spontaneous healing, and whether observation alone is an effective and safe form of management for selected small *M*. *ulcerans* lesions.
